# Retained dental roots of adults: A nationwide population study with panoramic radiographs

**DOI:** 10.1111/eos.12862

**Published:** 2022-04-01

**Authors:** Sanna Koskela, Miira M. Vehkalahti, Anna L. Suominen, Sisko Huumonen, Irja Ventä

**Affiliations:** ^1^ Department of Oral and Maxillofacial Diseases University of Helsinki Helsinki Finland; ^2^ Department of Oral and Maxillofacial Diseases Helsinki University Hospital Helsinki Finland; ^3^ Institute of Dentistry University of Eastern Finland Kuopio Finland; ^4^ Department of Oral and Maxillofacial Diseases Kuopio University Hospital Kuopio Finland; ^5^ Department of Public Health and Welfare Finnish Institute for Health and Welfare Helsinki Finland; ^6^ Diagnostic Imaging Center Kuopio University Hospital Kuopio Finland; ^7^ Research Unit of Oral Health Sciences University of Oulu Oulu Finland

**Keywords:** health surveys, panoramic radiography, third molar, tooth extraction, tooth root

## Abstract

The aim of this study was to assess the occurrence and nature of retained dental roots and their associations with demographics in the Finnish adult population. From the cross‐sectional nationwide Health 2000 Survey of the Finnish population aged 30 years and older, 6005 participants with clinical oral examination and panoramic radiographs were included. Occurrence and characteristics of all retained dental roots were examined. Statistical analyses included χ^2^, Kruskal–Wallis and Mann–Whitney U tests, and SAS‐SUDAAN calculations. The mean age of the 6005 participants (46% men and 54% women) was 53 (SD 14.6) years. At least one retained dental root was observed in 13% (*n* = 754) of the participants. The 1350 retained roots included 461 (34%) roots retained entirely in bone and 889 (66%) partly in bone. The most common location of a retained dental root was the third molar region. Occurrence of retained roots partly in bone was associated with male sex and lower education. Occurrence of retained third molar roots entirely in bone was associated with female sex, younger age, higher education, and living in a city. Among all retained dental roots, the preponderance of third molars emphasized the demanding nature of extracting the third molar in women.

## INTRODUCTION

Retained dental root is a diagnosis listed in the International Classification of Diseases 10th Revision (ICD‐10) as K08.3 [1]. Such roots occur when the crown has disappeared either during extraction or due to caries and the roots may locate inside the bone or may remain visible during clinical oral examination. The presence of retained roots is well‐documented in numerous studies of selected samples at dental care units. However, the occurrence of retained roots in population‐based studies is reported only for clinically visible root remains. The reason for this is that panoramic radiographs are rarely obtained in population studies. In a nationwide population‐based study from Finland in the 1980s, the prevalence of clinically visible retained dental roots was 15%, regardless of tooth type [2]. In that study of 7168 participants, the prevalence of retained roots was twice as great in men as in women.

Two earlier radiographic studies have focused on retained roots in larger samples. In a radiographic study from the 1960s, conducted at two university clinics in the USA on 2189 and 1685 participants, the occurrence of retained dental roots in edentulous and dentate participants together was 7.6% at age 21 and 37.8% at age 61 years or older [3]. Most of these roots were located in the posterior region of the jaws, and more were in the maxilla than in the mandible. An associated radiolucency was detected in 81% of the roots exposed to the oral cavity [3]. In another radiographic study from the 1960s, performed on 2000 Australian patients referred for removal of retained roots, 16.7% of the roots were exposed to the mouth, while 83.3% were located deeper [4]. However, these studies did not differentiate between tooth types or by their presence. There have been no larger studies on retained dental roots, and before the present study, none has used a representative sample with radiographs.

The aim of this study was to assess the occurrence and nature of retained dental roots and their associations with demographic features in the Finnish adult population. The underlying question is whether the examination of retained dental roots may reveal areas requiring attention in patient care.

## MATERIAL AND METHODS

### Study design and participants

This study was part of the Health 2000 Survey (BRIF8901, Bioresource Research Impact Factor) organized in 2000−2001 by the Finnish Institute for Health and Welfare [5]. This study is reported according to the Strengthening the Reporting of Observational Studies in Epidemiology (STROBE) guidelines. The survey was a nationally representative study that used a stratified two‐stage cluster sample of 8028 inhabitants aged 30 years and older [6, 7]. The sampling frame was regionally stratified according to five university hospital regions. From each of these stratums, 16 health center districts were sampled as clusters (*n* = 80). The final sampling units (inhabitants aged 30 years and older) were selected by systematic random sampling from each cluster. This design was used to obtain a sample that properly reflected the main demographic distribution of the Finnish population.

From the sample, a total of 6335 (79%) individuals participated in the clinical oral health examination [8]. Data on participants’ age, sex, and area of residence (city, town, or countryside) were extracted from the Population register of Finland. The level of education (basic, intermediate, or higher) was determined from an interview preceding the clinical phase [7].

The participants (*n* = 6335) in the clinical oral examination and the nonparticipants (*n* = 1693) did not differ by sex (*P *= 0.98). However, the participants were younger, were less likely to live in a city, and had higher level of education than the nonparticipants (*P* < 0.001; Mann–Whitney U and χ^2^ tests).

### Clinical and radiographic examination

The clinical oral health examination was performed in a portable dental unit by five calibrated dentists with assisting nurses [8]. All teeth, including third molars, were examined and a tooth was recorded as present if it was clinically visible or could be probed. A tooth was recorded as a retained dental root if more than half of all vertical surfaces of the crown were missing [8]. Quality assurance of clinical examinations included 2 weeks of training before commencement of the survey and both repeated and parallel measurements spread evenly throughout the field stage of the survey [8]. Related to dental status by tooth, consistency of parallel measurements with the reference dentist showed agreement in 93% of teeth, with a kappa value of 0.87 (95% CI 0.84–0.89) [8]. A participant's number of clinically recorded teeth was used to categorize the participants as being either clinically dentate or edentulous.

Immediately after the clinical oral examination of the 6335 individuals, panoramic radiographs were taken of 6115 voluntary participants. Digital panoramic radiography (Planmeca 2002 CC Proline) was performed with values of 58–68 kV and 4–10 mA depending on participant's size. After exclusion of images that were inadequate in the third molar region, 6005 radiographs remained and were included in the analysis. The 110 participants whose panoramic radiographs were excluded, did not differ by sex from those included (*P *= 0.85; χ^2^ test). However, they had less teeth in the clinical oral examination than the included participants (*P* < 0.001; Mann‐Whitney U test).

The included participants (*n* = 6005) in the radiographic examination and the excluded or nonparticipants (*n* = 2023) did not differ statistically significantly by sex (*P *= 0.06). However, the included participants were younger, were less likely to live in a city (*P *= 0.003) and had higher level of education than the nonparticipants (*P* < 0.001; Mann–Whitney U and χ^2^ tests).

The radiographic occurrence of permanent and deciduous teeth and their condition (tooth status, see below) were determined by three specialists in oral radiology. The specialists were trained and calibrated beforehand [8]. For the interexaminer reliability at training stage, concerning the readability of the radiograph the same interpretation was reported for 98% of the cases with a kappa value of 0.96 [8]. In the actual study, intraexaminer diagnostic quality was monitored by having the same radiologist re‐examine an earlier image taken a day before or earlier, for every 30th radiograph [8]. Tooth status was classified as missing, impacted, root partly embedded in bone, root wholly embedded in bone, implant, caries, or none of the above (such as a healthy tooth) [7]. The criteria described above were used to determine the number of each tooth present in the radiographs.

### Statistical analysis

For greater generalizability and comparability of the results, weighting coefficients calculated by Statistics Finland were used to correct effects of nonresponse and oversampling people aged 80 years or older [8]. When the observational unit was a participant, sampling weights, clusters, and stratums were used in the analysis. SAS‐Callable SUDAAN software version 11.0.3. was used to allow for the complex sampling method and to obtain weighted estimates (with 95% confidence intervals) of the occurrence of participants with retained roots representative of all Finns aged 30 years and older. All routine statistical analyses were performed with IBM SPSS Statistics version 27.

Participants with all types of retained roots were first analyzed together, followed by participants with retained roots entirely in bone and partly in bone. After examination of the number of retained roots, it was also necessary to categorize the participants as those with retained roots of third molars and those with retained roots of other teeth. Participant age was categorized as 30−39, 40−49, 50−59, 60−69, and 70 years or older. Differences among various subgroups were evaluated using χ^2^ test for frequencies and Mann–Whitney U or Kruskal–Wallis tests for means of independent groups.

### Ethical considerations

This research was conducted in full accordance with ethical principles such as those of the World Medical Association and the Declaration of Helsinki. Participants provided signed informed consent and participated in the study entirely on a voluntary basis. Ethical approvals for the examinations in 2000 were obtained from the Ethics committee of the National Public Health Institute and the Ethics committee of Epidemiology and National Health in the Hospital District of Helsinki and Uusimaa. A safety license was granted by the Radiation and Nuclear Safety Authority of Finland (No.: 4969/L1/00). The present study was approved by the Finnish Institute for Health and Welfare.

## RESULTS

Of the 6005 participants in the radiographic examination, 46% were men and 54% women. Their mean age was 53 years (SD 14.6; median 51; range 30−97 years).

In the 6005 panoramic radiographs, at least one retained dental root was found in 13% (*n* = 754) of the participants, more often in men than women (14% vs. 11%; χ^2^ = 9.21; df = 1; *P *= 0.002) (Table 1). The mean age of the 754 participants with retained roots was 57 years (SD 14.3; median 55; range 30−95 years).

**TABLE 1:** Distribution of the 754 participants with retained roots classified according to sex

	Total N	Participants with retained roots					
	Men/Women	Men		Women		Both combined	
Age(years)	N/N	n	% (95% CI)	n	% (95% CI)	n	% (95% CI)
30‐39	653/689	53	8 (6; 10)	38	6 (4; 8)	91	7 (6; 8)
40‐49	703/786	98	14 (11; 17)	77	10 (8; 12)	175	12 (10; 14)
50‐59	647/692	95	15 (12; 18)	79	11 (9; 13)	174	13 (11; 15)
60‐69	418/497	69	17 (13; 21)	84	17 (14; 20)	153	17 (15; 19)
≥70	336/584	70	21 (17; 25)	91	16 (13; 19)	161	18 (16; 21)
Total	2757/3248	385	14 (13; 15)	369	11 (10; 12)	754	13 (12; 14)

The denominator for the percentage calculations is the number of men or women or both in the age category. Percentages are weighted values with their 95% confidence intervals (CI) making the estimates representative of the Finnish population aged 30 years and older.

Participants with retained dental roots were older than those without such roots (57 vs. 52 years; *P* < 0.001, Mann–Whitney U test) (Table 2). Retained dental roots entirely in bone occurred more frequently among women than men (65% vs. 35%), while retained dental roots partly in bone prevailed in men (71% vs. 29%; χ^2^ = 93.07; df = 1; *P* < 0.001) (Table 2). Participants with retained dental roots had a lower level of education and were less likely to live in a city than those without such roots (Table 2). Among the participants recorded clinically as edentulous (*n* = 824), retained roots were found in 13% (Table 2).

**TABLE 2:** Distribution of selected demographic and clinical characteristics according to the occurrence of retained dental roots among 6005 participants aged 30 years and older

		Retained roots: Participants with					
		At least one root entirely in bone^a^ *n* = 392		Only roots partly in bone *n* = 362		No retained roots *n* = 5251	
Variable	Levels	n	% (95% CI)	n	% (95% CI)	n	% (95% CI)
Age group (years)	30–39	41	10 (1; 19)	50	14 (4; 24)	1251	24 (22; 26)
	40–49	69	18 (9; 27)	106	29 (20; 38)	1314	25 (23; 27)
	50–59	84	23 (14; 32)	90	26 (17; 35)	1165	24 (22; 27)
	60–69	96	25 (16; 34)	57	16 (7; 26)	762	14 (12; 17)
	≥70	102	24 (16; 32)	59	15 (6; 24)	759	13 (11; 15)
Sex^c^	Men	134	35 (27; 43)	251	71 (65; 77)	2372	47 (45; 49)
	Women	258	65 (59; 71)	111	29 (21; 37)	2879	53 (51; 55)
Education^c^, ^d^	Higher	89	23 (14; 32)	49	14 (4; 24)	1576	30 (28; 32)
	Intermediate	93	24 (15; 33)	127	35 (27; 43)	1708	33 (31; 35)
	Basic	209	53 (46; 60)	185	51 (44; 58)	1947	37 (35; 39)
Area of residence^c^	City	214	55 (48; 62)	190	53 (46; 60)	3259	62 (60; 64)
	Town	69	18 (9; 27)	53	15 (5; 25)	737	14 (12; 17)
	Countryside	109	27 (19; 35)	119	32 (24; 40)	1255	24 (22; 26)
Clinical dentition^c^, ^e^	Dentate	292	77 (72; 82)	355	99 (98; 100)	4518	87 (86; 88)
	Edentulous	98	23 (15; 31)	5	1 (0; 3)	721	13 (11; 16)
No. of roots/ person^c^	1	310	79 (75; 83)	218	60 (55; 65)	0	
	2	47	12 (9; 15)	60	17 (13; 21)	0	
	3‐22	35	9 (6; 12)	84	23 (19; 27)	0	
Mean age (95% CI)^b^	Years	58.5	58; 61	53.3	53; 55	51.9	51; 52

The denominator in percentage calculations is the number of people with the given retained root condition. Percentages are weighted values with their 95% confidence intervals (CI) making the values representative for the Finnish population aged 30 years and older.

^a^ In addition to at least one retained root entirely in bone, 26 of these participants also had retained roots located partially in bone.

^b^ *P* < 0.001, Kruskal–Wallis test.

^c^ *P* < 0.001, χ2 test.

^d^ Level of education was not available for 22 participants.

^e^ Clinical number of teeth was not available for 16 participants.

### Number of retained roots

**FIGURE 1:**
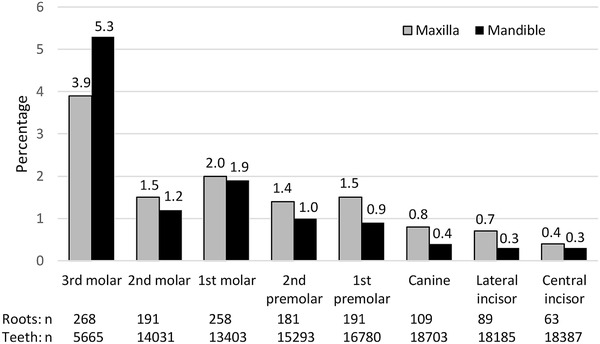
The proportion of retained dental roots (*n* = 1350) according to tooth type as observed in 6005 panoramic radiographs. The number of teeth observed indicates that many third molars are congenitally missing or have been extracted

Most participants with retained roots (84%, *n* = 635) had one or two retained dental roots; the highest number in a participant was 22 roots. The total number of retained dental roots counted over the 6005 radiographs was 1350; more were in the maxilla than the mandible (53% vs. 47%; χ^2^ = 13.49; df = 1; *P* < 0.001). Among all tooth‐like elements seen in panoramic radiographs, 1.2% comprised roots only (Figure 1). The most common location of a retained dental root was the molar region of either jaw, particularly the third molar region. The third molars accounted for 20% (*n* = 268) of all retained dental roots. When the two most common tooth types accounting for retained dental roots were compared (Figure 1), the proportion of third molar retained roots among all third molar teeth present in the radiographs was larger than that of the first molar roots (4.7% vs. 1.9%; χ^2^ = 116.9; df = 1; *P* < 0.001).

From the 1350 retained dental roots in panoramic radiographs, 461 (34%) were retained entirely in bone while the remaining 889 (66%) were partly in bone. Among the 461 retained roots entirely in bone, the most common location in the mandible was the third molar region (58%, *n* = 117), and in the maxilla, the first molar region (34%, *n* = 87). When both jaws were analyzed together, retained roots entirely in bone were more common in the third molar region than in other tooth regions (57% vs. 29%), while retained dental roots partly in bone occurred more often related to other teeth than the third molars (71% vs. 43%; χ^2^ = 75.74; df = 1; *P* < 0.001).

### Participants with third molar roots

Participants with retained roots of third molars alone were younger than participants with retained roots also in other dental areas (mean ages 52 vs. 58 vs. 58 years, respectively, in Table 3; Kruskal‐Wallis test = 27.12; df = 2; *P* < 0.001). Retained roots of teeth other than third molars were accumulated more often among older than younger age groups (Table 3).

**TABLE 3:** Distribution of the 754 participants with retained dental roots classified into three groups according to the tooth types affected

	Participants with retained roots of						
Age group (years)	Third molars alone		Both types		Other teeth alone		Total
	n	% (95% CI)	N	% (95% CI)	n	% (95% CI)	n
30–39	46	49 (39; 59)	3	3 (0; 7)	42	48 (38; 58)	91
40–49	41	23 (17; 29)	18	11 (6; 16)	116	66 (59; 73)	175
50–59	33	19 (13; 25)	15	9 (5; 13)	126	72 (65; 79)	174
60–69	26	17 (11; 23)	17	11 (6; 16)	110	72 (65; 79)	153
≥70	26	16 (10; 22)	13	8 (4; 12)	122	76 (69; 83)	161
Total	172	23 (20; 26)	66	9 (7; 11)	516	68 (65; 71)	754

The denominator for the percentage calculations is the number of people in the age category. Percentages are weighted values with their 95% confidence intervals (CI) making the estimates representative of the Finnish population aged 30 years and older

The prevalence of retained roots inside the bone was twice as great in women than men (Table 4). Participants with retained roots of third molars entirely in bone were younger, had higher level of education and were more likely to live in cities than participants with retained roots of other teeth in bone (Table 4). Most participants (91%, *n* = 357) with retained roots in bone had one or two such roots (Table 4).

**TABLE 4:** Distribution of selected demographic and clinical characteristics among the 392 participants with retained dental roots inside the bone classified into three groups according to the tooth types affected

		Participants with retained roots inside the bone					
		Third molars alone *n* = 131		Both types *n* = 17		Other teeth alone *n* = 244	
Variable		n	% (95% CI)	n	% (95% CI)	n	% (95% CI)
Age group^b^ (years)	30–39	37	28 (20; 36)	0	0	4	2 (0; 4)
	40–49	34	26 (18; 34)	1	6 (0; 17)	34	14 (10; 18)
	50–59	23	19 (12; 26)	4	26 (5; 47)	57	25 (20; 30)
	60–69	17	13 (7; 19)	6	36 (13; 59)	73	30 (24; 36)
	≥70	20	14 (8; 20)	6	32 (10; 54)	76	29 (23; 35)
Sex^c^	Men	39	32 (24; 40)	4	25 (4; 46)	91	38 (32; 44)
	Women	92	68 (60; 76)	13	75 (54; 96)	153	62 (56; 68)
Education^b^, ^e^	Higher	49	38 (30; 46)	1	7 (0; 19)	39	16 (11; 21)
	Middle	42	32 (24; 40)	0	0	51	21 (16; 26)
	Basic	40	30 (22; 38)	16	93 (81; 100)	153	63 (57; 69)
Area of residence^d^	City	76	59 (51; 67)	10	60 (37; 83)	128	53 (47; 59)
	Town	23	17 (11; 23)	1	6 (0; 17)	45	18 (13; 23)
	Country	32	24 (17; 31)	6	34 (12; 57)	71	29 (23; 35)
Clinical dentition^b^, ^f^	Dentate	117	91 (86; 96)	11	69 (47; 91)	164	69 (63; 75)
	Edentate	14	9 (4; 14)	6	31 (9; 53)	78	31 (25; 37)
No. of roots in bone/ person^b^	1	118	90 (85; 95)	0	0	192	79 (74; 84)
	2	8	6 (2; 10)	9	49 (25; 73)	30	13 (9; 17)
	3‐11	5	4 (1; 79)	8	51 (27; 75)	22	8 (5; 11)
Mean age^a^	Years	50.8	(48; 53)	64.8	(60; 70)	62.3	(61; 64)

The denominator in percentage calculations is the number of people with the given retained root condition. Percentages are weighted values with their 95% confidence intervals (CI) making the values representative for the Finnish population aged 30 years and older.

^a^ *P* < 0.001, Kruskal–Wallis test.

^b^ *P* < 0.001, χ^2^ test.

^c^ *P *= 0.270, χ^2^ test.

^d^ *P *= 0.485, χ^2^ test.

^e^ Level of education was not available for one person.

^f^ Clinical number of teeth was not available for two persons.

## DISCUSSION

The aim of this study was to assess the occurrence and nature of retained dental roots and their associations with demographic features in the Finnish adult population. The main finding was that the third molar region was the most frequent location for a retained dental root, both for all retained roots and for those retained entirely in bone. In addition, retained roots of third molars entirely in bone occurred more often in women than men. These findings add a new aspect to the extensively investigated third molar tooth.

A limitation of the study was that our data were two decades old. Even so, we decided to describe the retained dental roots of this unique population having data from panoramic radiographs, as so far, no such studies have been published. We presume that the prevalence of retained roots has not changed much during the 20 years. Another limitation of our study is the accuracy of the panoramic radiograph in detecting structures, especially in the anterior regions of both jaws and the maxilla [9]. However, in our study, the specialists in oral radiology interpreting the panoramic radiographs were calibrated beforehand and repeated assessments were made during reading [8]. A third limitation of our study was that the cross‐sectional data did not include information on the history of the teeth recorded as retained roots, such as the duration of being a retained root, the time since extraction, the experience of the clinician who extracted the tooth, or the method of anesthesia used during extraction. The etiology of retained roots has been alluded to in an Australian study on incomplete exodontia, where 89% of the 2000 patients had undergone the extraction more than 2 years ago (some up to 50 years ago) and only 22.5% of retained roots had caused symptoms or any demonstrable pathology [4].

The initial, nationally representative sample (*n* = 8028) were those who were invited to take part in the survey. Among these, 79% participated in the clinical oral examination and 76% in the panoramic radiography. Due to this exceptionally high response rate and the study design, analyzed samples can be considered nationally representative.

Among all teeth, it was surprising to observe the preponderance of retained third molar roots. In contrast, an earlier study from the 1960s showed that the maxillary molar region is the most frequent location for all types of retained roots [3]. A detailed study from 1981 showed that in edentulous jaws, the bicuspid and first molar regions in the maxilla predominate [10]. The difference between our findings and those of earlier studies may depend on the general improvement of oral and dental health since the 1960s. Consequently, among the few extracted teeth the third molar may be the most frequently extracted tooth with root fragments left behind, either purposefully or iatrogenically.

Our finding on the preponderance of the third molar root retention suggests that extraction of this tooth is a demanding procedure. Therefore, we wanted to determine whether there is any difference between retained roots of the two most common groups of retained roots, namely, the third and the first molar. The proportions (4.7% vs. 1.9%) of retained roots of all teeth present indicate that third molars were extracted more often than the first molars. However, in the mandible, but not in the maxilla, third molar roots were more often located wholly embedded in the bone than the first molar roots, which suggests that third molar roots in the mandible may fracture more easily.

Our study partly confirmed some earlier findings on the prevalence of retained dental roots of all teeth. The occurrence of 13% observed here for retained roots is lower than those of an earlier Finnish (15%), British (17%), and US (20%) studies [2, 3, 11]. The slightly lower occurrence of retained roots in our study may indicate that the level of oral and dental health has increased since the time of the oldest studies. Other factors may be improvements in instrumentation, technique, and education related to extractions.

Those who had only retained roots from third molars were younger on average than those with other locations of retained roots. This age difference may be explained by third molar extractions (and thus root fracture), which are most often performed in the age group of 20−40 years [12]. Another explanation may be the upward movement of third molar roots left behind, as demonstrated in coronectomy studies to occur over time [13], and therefore, in older persons this tendency to movement has brought some roots to the surface and subsequently the roots have been removed. The difference may be also associated with the surge of extraction of third molars beginning in the 1980s when all impacted teeth were indicated for removal [14].

Our findings suggest that roots may fracture more easily during tooth extraction in women than men. However, women may also be more likely to visit a dentist and have their third molars extracted. Earlier studies have not compared sexes for the presence of retained roots located entirely in bone. In a population study of clinically visible roots alone, a higher occurrence of retained roots in men was reported [2]. However, the sex‐difference was not apparent when using linear regression analysis [15].

An important aspect of retained dental roots is whether they need treatment [16]. Like most periradicular radiolucencies, carious root remnants are focuses of infection and clear indications for treatment. In our material, 6% of all participants had retained roots located partly in bone (Table 2), and therefore, suggestive of need of treatment. In the British study based on panoramic radiographs, 17% of the 1817 patients had retained roots [11]. However, according to the individual patient's dentist, only 5–7% of the patients needed treatment for the roots [11].

The coding for the diagnosis of a retained root in the ICD‐10 coding system [1] is confusing. It does not differentiate between the two types of retained roots, namely, those within bone after extraction and those exposed to the oral cavity and with caries. Therefore, separate codes could be added to the coding system for the two different types of retained roots. This would also clarify research on the subject of retained roots.

Our findings on the occurrence and characteristics of retained roots entirely in bone are likely to be applicable to all countries where oral and dental health is good, and third molars are the most frequently extracted teeth. Further research is needed on retained dental roots of third molars and the rate of complications around third molar removal to identify measures to reduce their occurrence.

In conclusion, although retained dental roots were not abundant in the population, the third molar region was the most frequently observed. This suggests that extraction of the third molar is challenging, especially in women. Our findings which take account of all retained dental roots are novel and further emphasize the distinctive character of the third molar tooth.

## CONFLICTS OF INTEREST

The authors report no conflicts of interest.

## AUTHOR CONTRIBUTION

**Conceptualization**: Sanna Koskela, Irja Ventä; **Methodology**: Sanna Koskela, Miira Vehkalahti, Irja Ventä; **Software**: Sanna Koskela, Miira Vehkalahti, Liisa Suominen, Irja Ventä; **Validation**: Liisa Suominen, Irja Ventä; **Formal analysis**: Sanna Koskela, Miira Vehkalahti, Liisa Suominen, Irja Ventä; **Investigation**: Sanna Koskela, Liisa Suominen, Sisko Huumonen, Irja Ventä; **Resources**: Planmeca; **Data Curation**: Miira Vehkalahti, Liisa Suominen, Sisko Huumonen; **Writing ‐ original draft preparation**: Sanna Koskela, Irja Ventä; **Writing ‐ review and editing**: Miira Vehkalahti, Liisa Suominen, Sisko Huumonen, Irja Ventä; **Visualization**: Irja Ventä; **Supervision**: Irja Ventä; **Project administration**: Finnish Institute for Health and Welfare; **Funding acquisition**: Finnish Dental Society Apollonia, Finnish Dental Association.
